# Institutional Questions

**Published:** 1899-08-19

**Authors:** 


### INSTITUTIONAL QUESTIONS.

**Furniture and Fittings.**

"Hospital Secretary" writes: Can you advise me where to obtain information as to ward furnishing on the most modern lines, and hints generally with regard to ward fittings? Where shall I find in London the best equipped and newest hospital buildings?

The very newest hospital in London is the just opened Royal London Ophthalmic Hospital, City Road, E.C., where the ward fittings have been carried out admirably, and everything is quite up-to-date. A new ward has recently been opened at St. Thomas's Hospital, and is in every particular a model of aseptic equipment, also possessing bath-rooms and lavatories which leave nothing to be desired. You will find the latest improvements and ward appliances at either of these institutions.

**Open-air Treatment of Consumption.**

"Chelmsford" writes: Is there any establishment for carrying out the open-air treatment of consumption in the Eastern Counties?

A sanatorium for this purpose is about to be started in Norfolk. Perhaps you know that for some months past one of the medical staff at the Norfolk and Norwich Hospital has made much use of this treatment in connection with the hospital.

**Poor Law Infirmaries.**

"A Guardian" writes: Can you tell me of any publication which gives some account of the development of the modern workhouse infirmary and hints on the management of such institutions, with general information on Poor Law matters?

You cannot do better than get Miss Louisa Twining's excellent little book, "Workhouses and Pauperism," one of the "Social Questions of To-day" series, published by Methuen and Co., Essex Street, W.C., price 2s. 6d. It is full of interesting and useful information, and should be possessed by every guardian. At the end of the book you will find a useful list of publications on Poor Law matters.

**North-Eastern Hospital for Children.**

"M. C." writes: Please tell me if a subscriber's letter is necessary to secure admission for a patient into the North-Eastern Hospital for Children, or what are the steps to be taken?

Write for a copy of the annual report to the secretary, at the City office, 27, Clement's Lane, E.C. In-patients are admitted by subscriber's free ticket or by payment of 2s. 6d. per week if the patient's friends are in a position to pay. Out-patients are admitted by subscriber's free ticket or by payment of 4d. on admission and 3d. a week afterwards, except in cases of extreme poverty, which are always treated gratuitously. Accident and emergency cases are attended to at all hours, medical cases every day between half-past twelve and half-past two, surgical cases on Wednesdays at this time, and on Fridays from half-past eight to ten a.m. Children over twelve are not eligible for treatment.

